# Genetic correlations between enteric methane and traits of economic importance in a beef finishing system

**DOI:** 10.1093/jas/skaf162

**Published:** 2025-05-13

**Authors:** Clodagh V Ryan, Thierry Pabiou, Deirdre C Purfield, David N Kelly, Craig P Murphy, Ross D Evans

**Affiliations:** Irish Cattle Breeding Federation, Ballincollig, Co. Cork, Ireland; Department of Biological Sciences, Munster Technological University, Bishopstown, Co. Cork, Ireland; Irish Cattle Breeding Federation, Ballincollig, Co. Cork, Ireland; Department of Biological Sciences, Munster Technological University, Bishopstown, Co. Cork, Ireland; Irish Cattle Breeding Federation, Ballincollig, Co. Cork, Ireland; Department of Biological Sciences, Munster Technological University, Bishopstown, Co. Cork, Ireland; Irish Cattle Breeding Federation, Ballincollig, Co. Cork, Ireland

**Keywords:** carcass traits, emissions, greenhouse gases, growth, terminal index

## Abstract

With the pressing global challenge of climate change, the potential to breed cattle that produce less lifetime methane offers a transformative solution that is both sustainable and impactful. The objective of this study was to determine the genetic correlations between enteric methane emissions and economically important traits included in the current terminal index used to breed animals for meat in Ireland. This terminal index is typical of terminal-type indexes used globally, constituting traits associated with calving performance, carcass merit, and efficiency traits such as feed intake and age at finish, as well as some ancillary traits such as docility. Methane and carbon dioxide flux measurements recorded from 2018 to 2024 using 10 GreenFeed Emission Monitoring systems in a progeny performance test center on 1,835 beef animals and a more expansive dataset from commercial farmers with phenotypic performance data on calving performance, carcass quality, and efficiency traits were available on up to 402,039 animals for analyses. Five trait definitions for methane and carbon dioxide emissions were derived: individual spot measures, 1-d, 5-d, and 10-d averages of spot measures, and a full test average per animal, where all emission measurements were averaged across the test period. (Co)variance components between all trait definitions and phenotypic performance traits were estimated using animal linear mixed models. Methane emissions were strongly correlated with feed intake ranging from 0.49 (standard error [**SE**] = 0.119) to 0.76 (SE = 0.057) and carcass weight ranging from 0.44 (SE = 0.050) to 0.50 (SE = 0.060) across trait definitions, suggesting that selection for reduced methane emissions could adversely impact growth and performance. An antagonistic correlation was found between methane and age at finish ranging −0.27 (SE = 0.063) to −0.18 (SE = 0.084), which suggests that animals who have an earlier finishing age produce more methane per day. Carcass conformation was positively weakly correlated with methane (0.09 to 0.12), thus suggesting there is a potential to select for improved carcass conformation with minimal impact on enteric methane emissions. Overall, these findings emphasize the need for breeding strategies that capture the trade-offs between reducing methane emissions and preserving economically valuable traits such as feed intake, carcass weight, and conformation in beef finishing systems.

## Teaser

This research provides critical insights into the genetic links between methane emissions and economically important traits in beef cattle. Elucidating the genetic relationship between methane emissions and economically important traits is necessary to determine the influence of current breeding strategies on emissions whilst providing a stepping stone for the incorporation of a methane trait into selection indexes.

## Introduction

Mitigation of climate change and ensuring the provision of food to a growing population are crucial goals of the 21st century (Fouts et al., 2022) and require innovative approaches across multiple sectors. In the agricultural sector, animal breeding has emerged as a leading mitigation strategy for reducing enteric methane emissions internationally due to changes being both permanent and cumulative (Pickering et al., 2015; Negussie et al., 2017; Lassen and Difford, 2020; de Haas et al., 2021). The potential to breed cattle that produce less methane offers a transformative solution that is both sustainable and impactful. To date, several studies have demonstrated enteric methane in cattle to be under moderate genetic control with considerable genetic variation, suggesting that breeding for reduced methane is most definitely possible (Oddy et al., 2014; de Haas et al., 2021; Manzanilla-Pech et al., 2021; van Breukelen et al., 2023; Ryan et al., 2024). Nevertheless, for methane to be incorporated into breeding goals, the relationship between methane and other economically important traits in a food production system must be elucidated. Selection for reduced methane without considering these relationships could lead to unintended antagonistic effects, similar to milk and fertility traits in dairy cattle (Pritchard et al., 2013) and methane and rumen size in sheep (Bain et al., 2014). An understanding of these genetic relationships is crucial for developing effective breeding programs that optimize environmental and economic outcomes simultaneously (Goddard and Hayes, 2009).

To date, only a limited number of studies in cattle have investigated the genetic correlation between methane emissions and production traits and their emphasis has primarily focused on the relationship with methane and feed intake (Lassen and Løvendahl, 2016; Zetouni et al., 2018; Breider et al., 2019; Robinson et al., 2020), and live weight and carcass traits (Donoghue et al., 2016, 2020; Duthie et al., 2017; Smith et al., 2021). The strong positive phenotypic and genetic relationship between feed intake and methane output, and by extension between carcass performance and methane, has been well established (Blaxter and Clapperton, 1965; Donoghue et al., 2016; Herd et al., 2014; Pelchen and Peters, 1998; Johnson et al., 2022; Ryan et al., 2024). Notably however, Ryan et al. (2024) demonstrated that the strength of these correlations was dependent on the methane trait definition used. Currently there is a lack of a consensus in the scientific community on the ideal methane trait definition for inclusion into breeding goals. Therefore, it is important to determine how the relationship with economically important traits varies across methane trait definitions in order to best determine the most appropriate trait for selection strategies. Furthermore, a significant gap in knowledge remains on the relationship between methane and non-growth related traits, such as calving performance and docility.

Therefore, the objective of the present study was to determine the genetic correlations between multiple methane and carbon dioxide trait definitions and economically important traits included in a terminal index for beef cattle. The index investigated was the current Terminal index used in Ireland and is typical of terminal-type indexes used globally, constituting traits associated with calving performance, carcass merit, and efficiency traits, as well as some ancillary traits such as docility (Kelly et al., 2020). Methane and carbon dioxide emission data from GreenFeed Emission Monitoring (**GEM**) systems on over 1,835 cattle destined for slaughter, as well as additional performance metrics such as feed intake and carcass performance were available. The results from this study provide the foundations for assessing the impact of selection for reduced enteric methane emissions on traits of economic importance in beef cattle.

## Materials and Methods

The data used in the present study were obtained from a pre-existing database managed by the Irish Cattle Breeding Federation (**ICBF**). Therefore, it was not necessary to obtain animal care and use committee approval in advance of conducting this study.

### Emission traits

#### Phenotypic data

The dataset used in this study is an expanded dataset of that previously described in detail by (Ryan et al., 2022, 2024). Briefly, enteric methane and carbon dioxide flux measurements were recorded between the years 2018 and 2024 using 10 GEM systems in the Gene Ireland Progeny Performance Test Centre (https://www.icbf.com/?page_id=12900) located in Tully, Co. Kildare, Ireland which operates as a commercial feedlot. Details of the enteric methane, carbon dioxide and feed intake measurements, as well as the diet fed and how the cattle were acclimatized to the GEM are described in detail elsewhere (Ryan et al., 2022). Measurements were recorded on 1,835 animals ranging from 356 to 897 d of age at test start date and consisted of 1,021 steers, 582 heifers, and 232 young bulls, with all animals slaughtered immediately after the end of test. All cattle were grouped in pens of 25 animals according to their sex, liveweight, and breed, and each group was referred to as a cohort. Cohorts comprised of animals of the same animal type, i.e., suckler bred beef, beef sired animals from the dairy herd and dairy bred animals. The breed breakdown of the animals included in this study, by sire and dam breed, are described in Supplementary Table S1.

The GEM systems used in this study were manufactured by C-Lock Inc. (C-Lock Inc., Rapid City, South Dakota). All GEM systems were calibrated at the start of each test period and machine settings are described elsewhere (Ryan et al., 2022). The test period ranged from 20 to 114 d in length and was the period of time where animals had full access to the GEM system post acclimatization. Test period length in the present study varied by cohort and consisted of a start date (i.e., when all animals in the pen had acclimatized to the GEM system) and an end date, immediately prior to slaughter. Test period length per cohort was determined by management decisions taken in the test station. Ancestry and genotypic data were available on all animals, and all animals included in the analysis were parentage and breed verified. Prior to filtering, a total of 454,445 GEM system individual spot measures were available from 1,835 animals destined for slaughter.

#### Data edits

Only enteric methane and carbon dioxide spot measures at least 2 min in duration were retained. Furthermore, the top 1% and bottom 1% of values for each emission trait were discarded (*n* = 9,723). The total number of visits per animal ranged from 1 to 453 visits (mean = 164.16, standard deviation = 83.42) throughout the test period and the total number of days each animal visited to the GEM system at least once ranged from 1 to 191 d. After edits, 286,193 GEM system individual spot measures were available on 1,794 animals.

#### Trait definition

Emission trait definitions were previously described by Ryan et al. (2024). Briefly, each individual emission measurement is reported as grams per day per spot measure, resulting in animals potentially having multiple methane and carbon dioxide grams per day values. Five trait definitions for both methane and carbon dioxide emissions were utilized in the present analysis; individual spot emission measures, 1, 5, and 10 d emission averaging periods where the individual spot measures were averaged within each respective time period and, lastly, a full test average per animal where all emission measurements were averaged across the test period for each animal (20 to 114 d on test). Each spot measure was assigned to 1 of 6 defined 4-h time periods across the day and in line with Ryan et al. (2024), 99% of animals in this study had at least 1 visit to the GEM system within each of the 6 defined time periods.

#### Statistical analysis

Univariate analysis for each methane and carbon dioxide trait definition was completed in DMU (Madsen and Jensen, 2013) using 3 statistical models similar to Ryan et al. (2024):

1) Full test average model:

**y** = X**b** + Z**a** + **e**

where **y** is the average phenotype across the duration of the test; **X** is the appropriate incidence matrix linking effects to the relevant animals; **b** is a vector of fixed effects which included the fixed class effects of contemporary group and the fixed regressions of breed composition, age, heterosis and recombination loss; **a** is a vector of random additive genetic effects with incidence matrix **Z,** and **e** is a vector of random residual values. Contemporary group included cohort number and GEM system number. Breed composition represented the proportion of each breed present in the animal and was based on the animals recorded ancestry from the ICBF database, with 14 breeds included (Angus, Aubrac, Belgian Blue, Charolais, Friesian, Hereford, Holstein, Jersey, Piedmontese, Parthenaise, Saler, Shorthorn, Simmental and Other). Heterosis and recombination loss were available from the ICBF database and calculated as per Van der Werf and De Boer (1989).

2) Multi-day average repeatability model:

**y** = X**b** + Z**a** + **e**

where **y** is the average phenotype across 1, 5, and 10 d of test; **X** is the appropriate incidence matrix linking effects to the relevant animals; **b** is a vector of fixed effects similar to the full test average model; **a** is a vector of random additive genetic effects with incidence matrix **Z** and **e** is a vector of random residual values. Contemporary groups for each multi-day (1-, 5-, 10-d) averaged phenotypes included the time period (day(s) of test) across which records were averaged, cohort number and GEM system number.

3) Spot measure repeatability model

**y** = X**b** + Z**a** + P**u + e**

where **y** is the spot measure phenotype; **X** is the appropriate incidence matrix linking effects to the relevant animals; **b** is a vector of fixed effects which included the fixed class effects of contemporary group and the fixed regressions of breed composition, age, heterosis and a time of day variable, where 6 classes were used for time of day, with each day split into 6 defined 4-h periods; **a** is a vector of random additive genetic effects with incidence matrix **Z**; **u** is a vector of random within-day permanent environmental effects, with incidence matrix **P** and **e** is a vector of random residual values. Contemporary group definition included cohort number, GEM system number and the date of record.

### Traits from national breeding objectives

The terminal index (Table S2) is the national breeding objective for beef cattle destined for slaughter within Ireland and comprises of 14 traits including: gestation, mortality, calving difficulty, docility, feed intake, age at finish, carcass weight, carcass conformation, and carcass fat, factory specification, carbon, polledness, and breed bonus. Calving difficulty is stratified into 2 traits, primiparous and multiparous calving difficulty as per Berry et al. (2019) and Berry and Ring (2020). The principle of the terminal index is based on low costs of production, i.e., low cost associated with calving, low mortality, short gestation, less feed consumed per kilogram of carcass, reduced age at finish, as high a return on the carcass as possible, and reduced carbon based on life cycle assessment. In short, the beef component of the terminal index estimates the economic value of an animal’s offspring based on live weight, carcass quality, and suitability for market. Genetic correlations between methane and carbon dioxide with 10 core traits of the terminal index (Table S2) were estimated using a series of bivariate animal linear mixed models. Four traits excluded from the analysis included: 1) the factory specification trait which is the likelihood trait of meeting the desired specifications at slaughter, and thus based on the carcass traits, 2) carbon which is a composite trait, whereby an additional economic weight was applied to the 10 core traits of the terminal index based carbon coefficients determined from a life cycle assessment methodology, 3) polledness, and 4) breed bonuses are applied to animals based on genotype.

Genetic correlations between the existing terminal index traits were estimated using Calo’s method (Calo et al., 1973) which calculates correlations based on estimated breeding values and reliability. Breeding values for all 10 core traits and their corresponding reliabilities were available from the ICBF database from July 2024 genetic evaluations. A cohort of 104 proven sires available for artificial insemination (**AI**) were used to estimate genetic correlations using Calo’s method; AI sires were required to have a minimum reliability of 0.95 in core terminal index traits, except feed intake, where a minimum reliability of 0.75 was required. A lower threshold was set for feed intake, as this trait is solely recorded through the Progeny Performance Test Centre, thus, there is a limitation on the number of animals that can be recorded; consequently, only 2 AI sires have achieved greater than 0.90 reliability. Calo’s genetic correlations across all traits were calculated using the following formula:


$$\begin{array}{l} r_{g}\;=\;\frac{\sqrt{\overset{n}{\underset{i=1}{\sum }}\rho_{i,EBV}*\;\;\;\overset{n}{\underset{i=1}{\sum }}\rho_{i,{EBV}^{\prime }}}}{\overset{n}{\underset{i=1}{\sum }}\rho_{i,EBV}*\rho_{i,{EBV}^{\prime }}}*\;r_{EBV,{EBV}^{\prime }} \end{array}$$

where ρ_*i*,EBV_ is the reliability of the estimated breeding value (EBV) for carcass weight for the bull *i*, ρ_*i*,EBV**’**_ is the reliability of the EBV for carcass conformation, and *r*_EBV,EBV’_ is the partial correlation between the 2 EBV adjusted for breed. This approach was systematically applied across all core terminal index traits.

### Calving data

Calving performance data for 7,975,559 calvings from the years 2010 to 2023 inclusive was available from the ICBF database. In Ireland, both multiparous calving difficulty and primiparous calving difficulty are subjectively scored by producers at the time of birth on a linear scale of 1 to 4 where 1 = no assistance, 2 = some assistance, 3 = considerable difficulty and 4 = veterinary assistance. For each calving event with a prior insemination event, gestation length was also estimated as the number of days from last recorded insemination to birth and only records between 271 and 300 d were retained for analysis. Furthermore, mortality data recorded as a binary variable if death occurred within 5 d of birth were also available from the ICBF database on 6,504,206 animals.

Contemporary group for each calving performance trait was defined as herd-year-season of calving generated using an algorithm as described in Berry and Evans (2014) and Berry et al. (2013) across the 4 seasons of the year. Only contemporary groups with 1) a minimum of 30% of animals which were half siblings to animals with GEM system measurements and 2) had a minimum incidence of 15% calving difficulty (calving score > 1) from 6 or more primiparous animals, or a minimum incidence of 15% calving difficulty (calving score > 1) from 12 or more multiparous animals were retained for the analysis of calving difficulty. This resulted in 33,409 and 90,948 calving events available for analysis of primiparous calving difficulty and multiparous calving difficulty, respectively. Similarly for gestation and mortality, only contemporary groups where a minimum of 20% of animals which were half siblings to animals with GEM system measurements and had a minimum mortality incidence rate of 5% from 10 or more animals within a group were retained. This mortality incidence is higher than the official mortality rate of 1.6% (Department of Agriculture Food and the Marine, 2021), however, mortality at birth tends to be under-reported with Mee (2020) reporting mortality rates ranging from 2% to 10% internationally. After edits, 402,039 mortality records and 17,075 gestation records were available for the bivariate analysis.

### Docility data

Docility data, recorded as part of routine linear scoring, was available from the ICBF database for 1,328 animals with GEM system records. Docility was subjectively measured by trained technicians, and scored on a scale from 1 to 10, where 1 equated to very poor docility, 5 equated to average docility and 10 equated to very docile. Linear scoring was conducted once per cohort, with all animals in the cohort recorded on the same day.

### Feed intake data

Feed intake data, as previously described in detail elsewhere (Ryan et al., 2022, 2024), was available on 1,794 animals. Briefly, each pen was equipped with 10 automatic feed stations (RIC Feed-Weigh Trough, Hokofarm Group BV, Marknesse, The Netherlands). Each station provided ad libitum access to feed. Acclimatization for feed intake was deemed complete when animals had spent between 21 and 30 d using the feed intake boxes. From Monday to Friday, the feed stations were refilled between approximately 0900 and 1700 hours, while on Saturday and Sunday, the feed stations were refilled between approximately 0830 and 1300 hours. Each pen also had ad libitum access to clean, fresh water via 5 water troughs spaced evenly between the automatic feed stations. Feed consumed through the GEM system was also included in daily feed intake; the concentrated offered within the GEM system was the same as that offered in the intake boxes and had a metabolizable energy concentration of 14.1 MJ/kg DM. Feed intake records were summed to a daily level of feed intake and expressed in kilograms of dry matter consumed.

Only animals with GEM system measurements had their feed intake data retained for analysis. Quality control metrics were applied to feed intake records by using a feeding rate metric (i.e., weight of feed consumed divided by the duration of visit) with the top and bottom 0.5% removed from the analysis (Kelly et al., 2020). Top and bottom 1% feed intake values based on the weight of feed consumed daily were also removed. This resulted in 130,525 daily feed intake records available for analysis on 1,794 animals.

Although not directly included in the current national breeding goal, feed conversion efficiency was incorporated in this analysis to help elucidate the relationship between emission traits and performance when expressed relative to liveweight growth. Simple linear regression was fitted through the serial liveweight measures of each individual animal separately to estimate a model intercept and average daily gain (i.e., linear regression coefficient) per animal. Only animals with at least 3 liveweight records after entering the test station were included in the calculation of average daily gain; 1,552 animals had ≥3 liveweight records. Feed conversion efficiency was then calculated as the ratio of average daily gain to daily dry matter intake. This resulted in 181,732 feed conversion efficiency records available on 1,552 animals. Feed conversion efficiency values were calculated to align with each emission trait definition, whereby daily values were matched to the spot measure and 1-d average emissions traits, and averaged over the corresponding 5-d, 10-d, and full test periods for use with multi-day and full test average emission traits.

### Carcass performance

Carcass information was available on 12,432,618 cattle from the years 2008 to 2023 from the ICBF database. Carcass weight was recorded on average, 2 h postslaughter following the removal of the head, legs, thoracic and abdominal organs, internal fats, and hide. Carcass conformation and carcass fat grade were scored on the 15-point EUROP classification system from a video image analysis of each carcass from a mechanical grading system (Pabiou et al., 2011); a carcass conformation score of 1 and a carcass fat score of 1 represents a poorly conformed carcass with little fat cover while a carcass conformation score of 15 and a carcass fat score of 15 represents an excellently conformed carcass with excess fat cover.

Only animals with a carcass weight record between 200 kg and 550 kg for steers and bulls, and 180 kg and 550 kg for heifers, a minimum slaughter age of 14 mo and a maximum of 30 mo were retained for analysis. For all slaughter traits, animals were grouped based on date of slaughter, herd prior to slaughter and sex (i.e., heifer, steer, and young bull). Contemporary group was defined as herd-year-season-sex of slaughter generated using an algorithm as described in Berry and Evans (2014) and Berry et al. (2013). To ensure uniform phenotypic variation across contemporary groups due to the large management effects, age at finish for each animal was calculated as the animal’s age at slaughter adjusted for the heterogeneity of variance within their respective contemporary group using the following model:


$$\begin{array}{l} y=\left(\left(\frac{x\;\;\;-\;\;\;\mu_{CG}}{SD_{CG}}\right)*SD\right)+\;\mu_{CG}\; \end{array}$$

where **y** was the adjusted age at finish; **x** was the phenotypic age at slaughter; **μ**_**CG**_ was the mean age at slaughter of the contemporary group; **SD**_**CG**_ was standard deviation age at slaughter of contemporary group and **SD** was the standard deviation of age at slaughter across the dataset. Only contemporary groups with a minimum of 15 animals, of which 30% of animals were half siblings to animals with GEM system measurements, were retained for analysis. This resulted in 148,428 slaughter records available for the bivariate analysis.

### Statistical analysis

The genetic correlations between methane and carbon dioxide traits with each of the 10 traits included in the terminal index were estimated usings a series of bivariate animal linear mixed models in DMU (Madsen and Jensen, 2013). The model fitted for each trait was:

**y** = X**b** + Z**a** + **e**

**w** = X**b** + Z**a** + **Pu **+ **e**

**v **= X**b** + Z**a** + Q**d** + **e**

**t** = X**b** + Z**a** + **e**

where **y** is the slaughter phenotype (carcass weight, carcass conformation, carcass fat, age at finish); **w** is the feed intake phenotype (feed intake, feed conversion efficiency); **v** is the calving phenotype (primiparous calving difficulty, multiparous calving difficulty, gestation, mortality); **t** is the docility phenotype; **X** is the appropriate incidence matrix linking effects to the relevant animals; **b** is a vector of fixed effects which included the fixed class effects of contemporary group and the fixed regressions of breed composition, heterosis and recombination loss for all traits; **a** is a vector of random additive genetic effects with incidence matrix **Z**; **u** is a vector of random between-day permanent environmental effects, with incidence matrix **P**; **d** is a vector of random dam genetic effects with incidence matrix **Q** and **e** is a vector of random residual values. Additional fixed effects were included in vector **b** dependent on trait: age at slaughter was included for carcass weight, carcass conformation and carcass fat analysis; carcass weight and carcass fat were included for age at finish analysis; age was included for both feed intake and docility analysis; technician was included for docility analysis; dam age and sex were included for both primiparous and multiparous calving difficulty analysis; dam parity was included for multiparous calving difficulty, gestation and mortality analysis and a fixed effect for the interaction between dam age and dam parity was included for gestation and mortality analysis. Breed composition represented the proportion of each breed present in the animal, with 14 breeds included (Angus, Aubrac, Belgian Blue, Charolais, Friesian, Hereford, Holstein, Jersey, Piedmontese, Parthenaise, Saler, Shorthorn, Simmental and Other). In all bivariate analyses, with the exception of feed intake traits, residual (co) variance between the methane trait and the index trait was fixed to zero, as animals did not have a methane measurement at the time of recording of that trait. For the bivariate analysis between feed intake and spot measure emissions, the residual (co) variance was estimated between the last emission measurement of the day and the corresponding day of feed intake data. For 1-d average emissions, residual (co) variance was estimated for the corresponding day of feed intake. Residual (co) variance was not estimated for 5-d, 10-d or full test average methane due to an inability to attribute the emission phenotype to an appropriate feed intake record. Feed conversion efficiency was treated using the same residual structure as feed intake for spot measure and 1-d average emission traits, whereby residual (co) variance was estimated using the corresponding daily record. However, for feed conversion efficiency models involving 5-d, 10-d, and full test average methane traits, residual (co) variance was estimated rather than fixed to zero, as both the feed conversion efficiency and methane traits were derived from performance over the same test period and therefore could be considered contemporaneous.

In all models, the relationships among animals were accounted using the numerator relationship matrix using 5 generations of pedigree information, where available. The coefficient of variation (**CV**) was calculated for all traits using the following formula: $CV=\;\frac{\sigma }{\mu }*100$, where σ is the standard deviation of the trait and μ is the average.

## Results and Discussion

Summary statistics for all traits included in the study are in Table 1 and 2. Depending on the trait definition, mean methane emissions varied from 238.5 to 241.6 g/d, and the CV ranged from 21.9% for the full test average methane trait definition to 36.5% for spot measure methane (Table 1). Despite the increase in population size, heritability estimates across all trait definitions were similar to that previously reported by Ryan et al. (2024). Heritability estimates for methane emissions were moderate and ranged from 0.15 (SE = 0.024) for the spot measure trait to 0.39 (SE = 0.086) for the full test average methane trait. Internationally, similar moderate methane heritabilities have been reported, with estimates varying depending on the methane measurement tool, trait definition, and number of records. Pszczola et al. (2017) reported methane heritability estimates of 0.23 to 0.30, Lassen and Løvendahl (2016) reported 0.21, Breider et al. (2019) reported 0.12 to 0.45, and van Breukelen et al. (2023) reported 0.19 and 0.33, all of which were estimated in dairy cow populations, whereas Donoghue et al. (2016) reported a heritability of 0.27 in beef cattle. Irrespective of the trait definition and measurement approach, these results corroborate that methane is under moderate genetic control and ample genetic variation exists (Table 1) to select for reduced daily methane emissions through genetic selection.

**Table 1.** Summary of GreenFeed emission including mean, standard deviation (SD), minimum (min.), maximum (max.), coefficient of variation (CV), heritability (*h*^2^), standard error of heritability (SE) and genetic variation (σ_g_)

	Trait	Number of animals	Number of records	Mean (SD)	Min	Max	CV	*h* ^2^ (SE)	σ_g_
Methane	Spot measure	1,794	286,193	241.6 (88.11)	48.3g	511.0g	36.5%	0.15 (0.024)	27.24
	1-d average	1,794	89,966	240.5 (71.00)	48.4g	510.7g	29.5%	0.15 (0.033)	20.74
	5-d average	1,794	20,393	239.2 (61.53)	48.4g	490.4g	25.7%	0.25 (0.054)	21.29
	10-d average	1,794	10,971	239.0 (58.93)	48.4g	469.8g	24.7%	0.29 (0.062)	21.27
	Full test average	1,794	1,794	238.5 (52.29)	72.3g	411.3g	21.9%	0.39 (0.086)	21.25
Carbon dioxide	Spot measure	1,794	286,193	9,514.4 (1,854.86)	4,718.7g	14,757.2g	19.5%	0.32 (0.032)	824.21
	1-d average	1,794	89,966	9,526.6 (1,529.64)	4,726.6g	14,756.5g	16.1%	0.24 (0.044)	531.26
	5-day average	1,794	20,393	9,535.7 (1,384.85)	4,807.9g	14,664.5g	14.5%	0.36 (0.065)	543.49
	10-day average	1,794	10,971	9,543.4 (1,349.11)	4,853.7g	14,473.0g	14.1%	0.41 (0.073)	553.64
	Full test average	1,794	1,794	9,459.6 (1,294.38)	4,853.7g	12,555.3g	13.7%	0.50 (0.092)	555.70

**Table 2.** Summary of available data from terminal index traits including mean, standard deviation (SD), minimum (min.), maximum (max.), coefficient of variation (CV), heritability (*h*^2^), standard error of heritability (SE) and genetic variation (σ_g_)

	Trait^*^	Number of animals	Number of records	Mean (SD)	Min.	Max.	CV	h^2^ (SE)	σ_g_
Calving	Gestation	17,075	17,075	280.5 (5.88)	271.0	300.0	2.1%	0.55 (0.031)	3.39
	Mortality	402,039	402,039	0.079 (0.27)	0.0	1.0	341.7%	0.03 (0.004)	0.05
	Primiparous calving difficulty	33,409	33,409	1.5 (0.76)	1.0	4.0	50.6%	0.27 (0.046)	0.39
	Multiparous calving difficulty	90,948	90,948	1.4 (0.65)	1.0	4.0	46.4%	0.15 (0.019)	0.25
	Docility	1,328	1,328	7.5 (1.07)	2.0	10.0	14.2%	0.52 (0.104)	0.68
Efficiency	Feed intake	1,794	130,525	11.8 (2.61)	3.8	25.0	22.1%	0.22 (0.044)	0.90
	Age at finish	148,428	148,428	711.3 (103.45)	356.6	960.2	14.5%	0.25 (0.012)	13.13
Carcass	Carcass weight	148,428	148,428	317.6 (48.48)	180.0	550.0	15.3%	0.37 (0.013)	16.10
	Carcass conformation	148,428	148,428	5.1 (1.96)	1.0	15.0	38.4%	0.38 (0.013)	0.59
	Carcass fat	148,428	148,428	7.8 (1.88)	1.0	15.0	24.1%	0.31 (0.012)	0.69

^*^units of measurement: Gestation, days; Mortality, percentage; Primiparous calving difficulty, producer recorded scale (1 to 4); Multiparous calving difficulty, producer recorded scale (1 to 4); Docility, technician recorded scale (1 to 10); Feed Intake, kilograms of dry matter; Age at Finish, days; Carcass Weight, kilograms; Carcass Conformation, processor recorded scale (1 to 15); Carcass Fat, processor recorded scale (1 to 15).

### Calving traits

To our knowledge, no genetic correlations between methane and calving traits have been previously estimated in beef populations. A moderate negative genetic correlation ranging from −0.17 to −0.24 (Table 3) was estimated between all methane trait definitions and gestation length, suggesting that animals with shorter gestation lengths produced more daily methane. Richardson et al. (2021) also reported a negative correlation, albeit weaker (−0.13) between a greenhouse gas index, constructed using methane coefficients, and gestation length in 3,412 Australian Holstein bulls. It is important to note that although the estimated genetic correlations in the present study were adjusted for breed, breed effects may have played a role in the strength of the correlation between methane and gestation length; substantial genetic variation existed for gestation length across the breeds, ranging from −3.56 d for Friesian to +13.66 d for Blonde d’Aquitaine. Nevertheless, as a reduced gestation length is currently favorable in the terminal index breeding goal, thus, the negative relationship between methane and gestation length suggests that the current selection strategy could inadvertently lead to increased methane emissions and presents a potential conflict between optimizing for calving efficiency and minimizing environmental impact. In comparison, the positive correlation between methane and mortality (ranging from 0.32 to 0.37; Table 3) suggests that continuation of current selection decisions (for reduced mortality) would give rise to reduced methane emissions. However, it is important to acknowledge that the mean mortality incidence in the present dataset was 7% (Table 2); comparably higher than national incidence rates (1.6% (Department of Agriculture Food and the Marine, 2021), which are most likely under-reported due to “farm-blindness” (Mee, 2020).

**Table 3.** Genetic correlations between methane traits and economically important traits in a finishing system, with standard error (SE) in parenthesis

Index trait		Methane Trait				
		Spot measure (SE)	1-d average (SE)	5-d average (SE)	10-d average (SE)	Full test average (SE)
Calving traits	Gestation	−0.17^*^ (0.026)	−0.24^**^ (0.083)	−0.23^**^ (0.082)	−0.24^**^ (0.081)	−0.23^**^ (0.083)
	Mortality	0.37^**^ (0.128)	0.34^***^ (0.147)	0.32^***^ (0.145)	0.33^***^ (0.146)	0.34^***^ (0.146)
	Primiparous calving difficulty	0.23 (0.160)	0.15 (0.184)	0.13 (0.181)	0.13 (0.182)	0.14 (0.188)
	Multiparous calving difficulty	−0.12 (0.158)	−0.09 (0.147)	−0.09 (0.145)	−0.13 (0.145)	−0.10 (0.149)
	Docility	0.22^**^ (0.073)	0.21^**^ (0.073)	0.22^**^ (0.071)	0.22^**^ (0.074)	0.21^**^ (0.074)
Efficiency traits	Feed intake	0.57^*^ (0.097)	0.49^*^ (0.119)	0.51^*^ (0.123)	0.50^*^ (0.124)	0.76^*^ (0.057)
	Age at finish	−0.27^*^ (0.063)	−0.27^**^ (0.083)	−0.28^*^ (0.081)	−0.23^**^ (0.081)	−0.18^***^ (0.084)
Carcass traits	Carcass weight	0.44^*^ (0.050)	0.50^*^ (0.060)	0.49^*^ (0.059)	0.48^*^ (0.059)	0.49^*^ (0.061)
	Carcass conformation	0.09 (0.060)	0.11 (0.072)	0.10 (0.070)	0.11 (0.070)	0.12 (0.071)
	Carcass fat	0.32^*^ (0.056)	0.39^*^ (0.068)	0.39^*^ (0.066)	0.39^*^ (0.067)	0.38^*^ (0.069)

Significance levels are denoted as follows: ^*^P < 0.001, ^**^P < 0.01, ^***^P < 0.05.

The negative correlation observed between methane and gestation length may reflect underlying differences in metabolic activity or feed intake associated with prepartum and postpartum early-life growth potential (Gore et al., 1994), both of which may contribute to increased methane production. Likewise, the positive correlation between methane and mortality may be related to differences in rumen development or microbial composition, potentially affecting energy availability or immune function in early life (Du et al., 2023). Further research perhaps incorporating microbiome and rumen physiology data may help elucidate these underlying biological mechanisms.

Calving difficulty is a key driver of farm profitability in both dairy and beef systems, with cases of calving difficulty being associated with reduced animal productivity, veterinary costs, increased labor costs and increased risks of both dam and calf mortality (Martin-Collado et al., 2017). In Ireland, primiparous and multiparous calving difficulty are evaluated separately since 2020 to allow farmers to make more accurate breeding decisions (Evans, 2020). In the current study, primiparous calving difficulty had an estimated heritability of 0.27 (SE = 0.045), whereas multiparous calving difficulty had a heritability estimate of 0.15 (SE = 0.019) with both estimates aligning with the literature for calving difficulty (0.34, Burfening et al., 1978; 0.27; Phocas and Laloë, 2003; 0.15; Phocas and Sapa, 2004; Ahlberg et al., 2016; 0.32). Primiparous calving difficulty had a weak to moderate positive genetic correlation with methane (0.13 to 0.23), but conversely, multiparous calving difficulty had a weak negative correlation (−0.09 to −0.13) (Table 3). Neither correlation was statistically significant for any methane definition (Table 3). The negative correlation with methane in multiparous animals may be a reflection of the relationship between animal size and calving difficulty; multiparous animals are typically larger than primiparous animals (Berry and Evans, 2022) and, thus, have lower incidence of calving difficulty (Table 2), in return larger animals have been shown to phenotypically produce more methane (Herd et al., 2014; Ryan et al., 2022). Overall, these correlations suggest a weak to moderate unfavorable relationship may exist between calving performance and methane emissions, with animals genetically predisposed to produce more methane tending to have shorter gestation lengths and, in primiparous animals, a higher likelihood of calving difficulty. However, this should be interpreted with caution, as shorter gestation lengths are generally associated with lower birth weights and reduced calving difficulty (Everett and Magee, 1965). The observed correlations may be influenced by indirect effects or confounding variables not fully accounted for in the current dataset. A larger dataset may help to clarify these associations and determine whether they reach statistical significance.

### Docility

Genetic correlations between docility and methane were consistent across all methane trait definitions, ranging from 0.21 to 0.22 with SE of 0.07 for all traits (Table 3). A moderate positive correlation between methane and docility suggests that more docile animals produce more methane. Previous work by Burrow and Dillon (1997) reported that more docile animals exhibit increased daily weight gain and this is corroborated by Sims et al. (2023). In the present study, positive correlations were found between both carcass weight and docility (0.15), and feed intake and docility (0.09) (Table 4). Therefore, the positive relationship between methane and docility may, in part, reflect the underlying positive correlations between docility and both carcass weight and feed intake, particularly given the well-established strong relationship between feed intake and methane emissions (Donoghue et al., 2016; Ryan et al., 2024).

**Table 4.** Calo’s correlations between terminal index traits

Index Trait		Calving traits				Docility	Efficiency traits		Carcass traits	
		Gestation	Mortality	Primiparous calving difficulty	Multiparous calving difficulty		Feed Intake	Age at Finish	Carcass Weight	Carcass Conformation
Calving traits	Mortality	0.09								
	Primiparous calving difficulty	0.22	0.70							
	Multiparous calving difficulty	0.19	0.71	0.97						
	Docility	0.03	0.08	−0.03	0.05					
Efficiency traits	Feed intake	0.05	0.03	0.21	0.14	0.09				
	Age at finish	−0.25	−0.03	−0.25	−0.23	−0.01	−0.51			
Carcass traits	Carcass weight	0.00	0.26	0.41	0.44	0.15	0.42	−0.29		
	Carcass conformation	0.02	0.34	0.31	0.33	−0.09	−0.10	−0.03	0.47	
	Carcass fat	0.14	−0.23	−0.13	−0.15	−0.04	0.11	−0.36	−0.27	−0.23

### Feed intake

**Figure 1.**
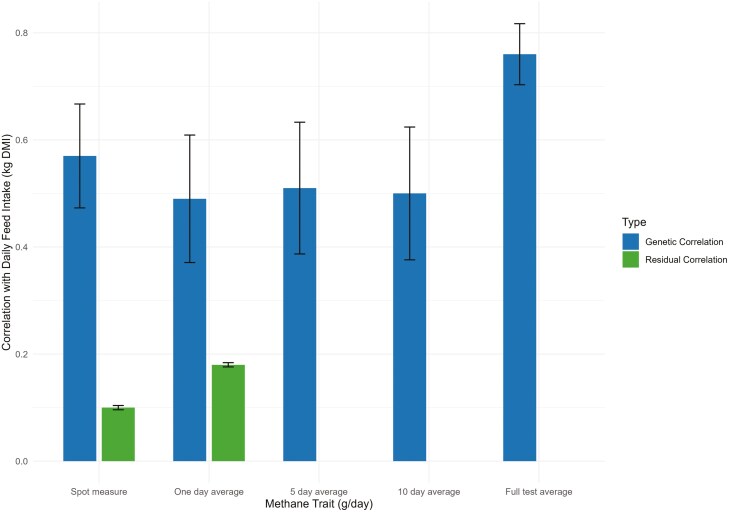
Genetic and residual correlations between daily feed intake (kg dry matter intake) and methane traits (g/d) with error bars. Error bars represent the standard error of correlation coefficients.

As expected, feed intake had a strong, positive genetic relationship with all definitions of methane, ranging from 0.49 (SE = 0.119) for 1 d average methane trait definition to 0.76 (SE = 0.057) for full test average methane (Table 3). Where possible, the residual correlation between daily feed intake and methane was estimated, resulting in a residual correlation of 0.10 and 0.18, for spot measure methane and 1 d average methane, respectively (Figure 1). These low to moderate residual correlations highlight that some of the relationship between methane and feed intake remains to be captured by the current models. One possibility for these residuals may be that the rumen microbiome, which is partially controlled by host animal genetics, is unaccounted for in the current analysis. Difford et al. (2018) and Roehe et al. (2016) demonstrated host animal genetics influenced a moderate amount of variation in rumen microbiome and, ultimately methane production. Furthermore, other factors such as lifetime diet and environmental conditions are also likely contributors to the unexplained variation in methane production (Hristov et al., 2013; Yáñez-Ruiz et al., 2015; Clemmons et al., 2019; Arshad et al., 2021).

The genetic correlations between both spot measure methane and full test average methane with feed intake in the present study differ to that previously reported by Ryan et al. (2024) whereas estimates for 1-, 5-, and 10-d averages were similar. The lower correlations for spot measure methane estimates in the present study may be due to the increased volume of feed data and less stringent data editing, allowing more feed intake data in the current analysis (*n* = 130,525) compared to Ryan et al. (2024) (*n* = 100,260). Additionally, the current study opted for a repeatability model for daily feed intake to better capture variation in feed intake across the test period whereas Ryan et al. (2024) derived the genetic correlation from feed intake records from days with corresponding GEM system records.

Many studies to date have determined that a moderate to strong genetic and phenotypic relationship between methane and feed intake exists in both beef and dairy cattle (*r*_g_ = 0.60, Difford et al., 2020; *r*_g_ = 0.84, *r*_p_ = 0.71, Donoghue et al., 2016; *r*_g_ = 0.42, *r*_p_ = 0.47, Manzanilla-Pech et al., 2021; *r*_g_ = 0.42, *r*_p_ = 0.49, Richardson et al., 2021; *r*_g_ = 0.49 to 0.72, *r*_p_ = 0.31, Ryan et al., 2022, 2024; *r*_p_ = 0.50, Smith et al., 2021). The strong genetic correlation that exists indicates a clear potential for simultaneous improvement in both feed efficiency, which is economically relevant, and reduced methane output, creating a beneficial ‘win-win’ scenario, assuming minimal impact on carcass quality. Whilst methane currently does not yet have a direct impact on the profitability of finishing systems in Ireland, the production of enteric methane accounts for a 2% to 12% gross energy intake loss in cattle (Johnson and Johnson, 1995). A reduction in methane production, therefore, may reduce the gross energy intake loss, reduce overall feed cost and increase overall profitability (Manzanilla-Pech et al., 2022).

Feed intake is a valuable, yet costly trait to measure directly, and limited records are available in many breeding programs. This scarcity has driven the search for indirect measures that could serve as proxies for feed efficiency (Williams et al., 2019; Davison et al., 2021). The GEM system, which measures both methane and carbon dioxide, offers a practical phenotyping solution for breeders, particularly in settings where direct feed intake data is not available (Ryan et al., 2024). The strong genetic correlations between both methane, and carbon dioxide with feed intake implies breeders can select for feed efficiency using methane or carbon dioxide measurements as proxies, this is particularly advantageous as widescale methane phenotyping programs become more routine (de Haas et al., 2021).

Although not currently included in the Irish national breeding goal, feed conversion efficiency was included in the present analysis to further explore the biological relationship between animal efficiency and methane emissions. As a ratio of average daily gain to daily feed intake, feed conversion efficiency captures the animal’s ability to convert feed into live weight gain and therefore reflects both performance and intake in a single measure (Koch et al., 1963). While the terminal index accounts for these traits independently, with carcass weight representing revenue and feed intake representing cost, feed conversion efficiency provides additional, important, biological context. The heritability of feed conversion efficiency in the current study was low (0.09; SE = 0.067), consistent with expectations for ratio traits. Genetic correlations between feed conversion efficiency and methane ranged from −0.16 (SE = 0.159) for the 5-d average methane trait to −0.21 (SE = 0.159) for both spot and full test average methane (Table S3). Although the estimates were not statistically different from zero, the consistent negative direction across all methane definitions suggests a potential biological trend whereby more feed efficient animals may produce less methane per day. However, as a ratio trait, feed conversion efficiency presents interpretational challenges due to the interdependence of its component traits (Gunsett, 1984; Zetouni et al., 2017), and caution is required when using it to infer causality. While selection on feed conversion efficiency alone is unlikely to substantially reduce methane emissions, it may offer useful biological insight into the relationship between intake, growth, and methane production, particularly when emissions are expressed on an intensity basis.

### Carcass traits

The average (SD in parenthesis) carcass weight, fat, conformation score and age at finish were 317.6 kg (48.48 kg), 7.8 units (1.88 units), 5.1 units (1.96 units) and 711.3 d (103.45 d), with large phenotypic variation due to the differences between sexes and breeds included in the analysis (Supplementary Table 1). Overall, carcass output was positively correlated with methane, with carcass weight showing the strongest genetic correlations ranging from 0.44 (SE = 0.050) for the spot measure methane to 0.50 (SE = 0.060) for the 1-d average methane (Table 3). This corroborates previous phenotypic findings that animals with genetic predisposition for heavier carcass weights also tend to produce more methane (Smith et al., 2021; Ryan et al., 2022). This relationship may be explained by physiological differences, as larger animals selected for increased carcass weight have been shown to possess greater rumen capacity (Nutt et al., 1980). This has been further corroborated in sheep, where animals with larger rumens have been demonstrated to emit more methane compared to those with smaller rumens (Goopy et al., 2014).

To our knowledge, no research to date has elucidated the genetic relationship between methane and carcass conformation, however, positive correlations for analogous traits such as eye muscle area (0.40; Donoghue et al., 2016) and dairy cow conformation (0.11; Pszczola et al., 2019) have been previously reported. In the current study, carcass conformation and methane were weakly but positively correlated across all trait definitions (0.09 to 0.12; Table 3) suggesting that producers could potentially select for more conformed animals with negligible impact on methane emissions. This low correlation may be particularly beneficial when selecting for improved efficiency in dairy cattle where 30.3% of steers originating from dairy herds fail to meet the desired EUROP carcass conformation speciation of ≥O = (Kenny et al., 2020).

In comparison to carcass conformation, carcass fat was moderately positively correlated (ranging from 0.38 to 0.39) across all methane traits (Table 3). Previous work by Donoghue et al. (2016) using similar traits of rib and rump fat thickness also reported positive genetic correlations between methane and fatness of 0.11 (SE = 0.16) and 0.10 (SE = 0.15), respectively. This positive correlation suggests selection for reduced methane emissions, would also result in a reduction in carcass fat, and this in turn may aid in reducing producer feed costs as fat deposition requires 2.25 times the energy compared to muscle accretion (NRC, 2000). This would be of particular benefit to heifer finishing systems, where 21.2% of heifer carcass have been reported to exceed the desirable EUROP fat score of >4 = for processing (Kenny et al., 2020). Furthermore, some retail markets and beef processors penalize overfat carcasses (Fisher, 2007) as overfat carcasses costs some processors in terms of the labor and waste associated with trimming excess fat off the carcass (Kelly et al., 2019). Therefore, potentially reducing methane emissions and, consequently, reducing carcass fat could aid in increasing producer profitability, although we must be cognizant of the balance needed within a breeding goal to still achieve the desirable EUROP carcass fat cover of between 2+ and 4 = remains.

**Figure 2.**
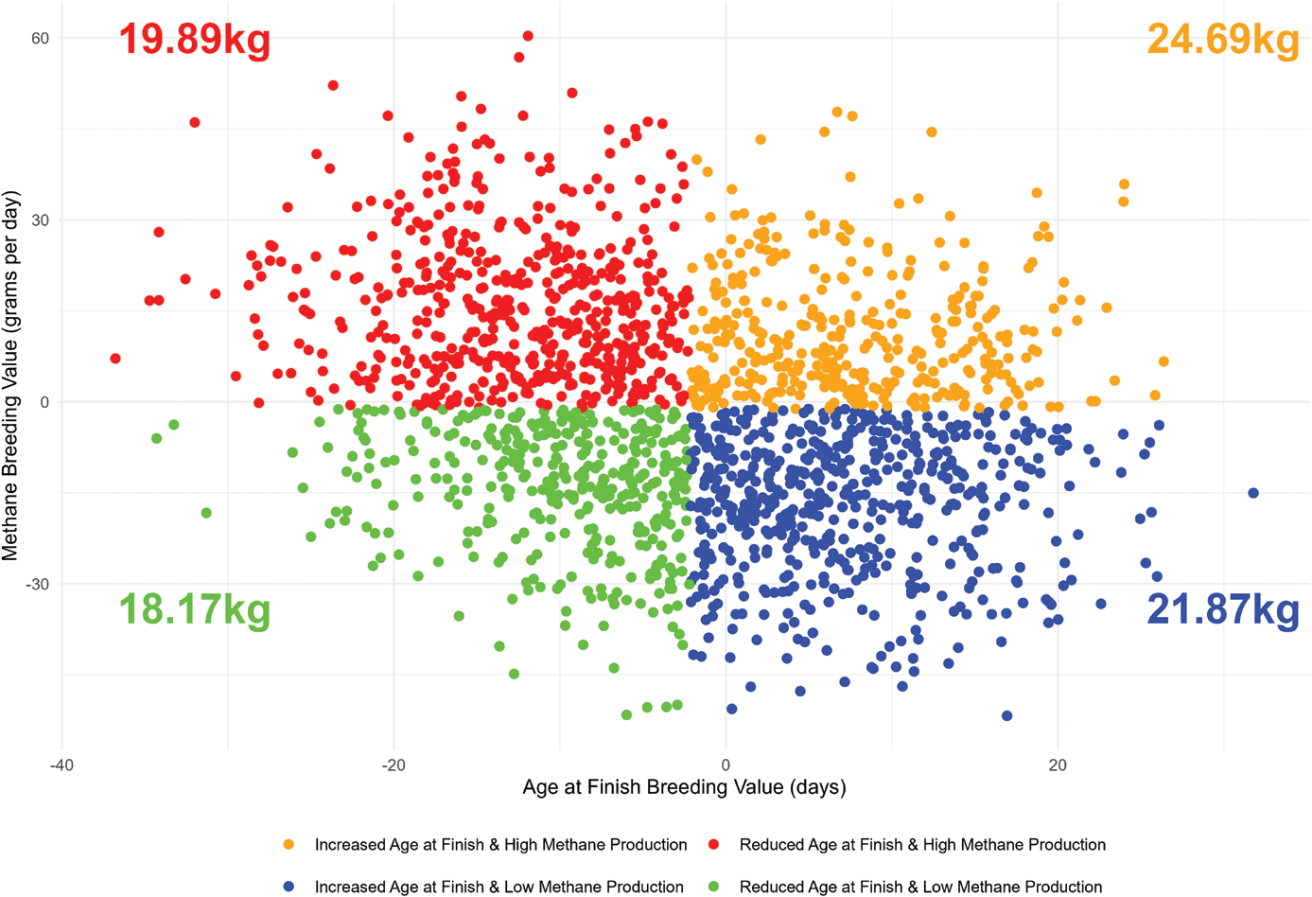
Relationship between breeding values for age at finish and methane emissions in animals with a methane phenotype, including the total predicted methane for each quartile in the finishing period.

Reducing the finishing age of animals in the national herd has been recognized in both the Irish Climate Action Plan (Government of Ireland, 2024) and the Teagasc Marginal Abatement Cost Curve (Schulte et al., 2012) as a cost-effective measure to reduce agricultural greenhouse gas emissions (Crosson et al., 2024). This approach has also garnered attention elsewhere, with similar strategies being explored and implemented in countries such as the United Kingdom, New Zealand, Australia, and across the European Union (Berndt and Tomkins, 2013; Bell et al., 2021; Stevens et al., 2023; Sabia et al., 2024). The correlation between age at finish and methane varied across methane definition, albeit all correlations were moderately negative, and ranged from −0.18 ± 0.084 for full test average methane to −0.28 ± 0.081 for 5-d average methane (Table 3). The antagonistic relationship between age at finish and methane poses a difficulty for methane mitigation strategies, as it suggests that animals that finish earlier produce more methane on a daily basis, as the methane traits are expressed in grams per day. This negative relationship may be linked to the metabolic and dietary requirements of faster-growing animals, which consume more feed daily to support accelerated growth (Berry and Pryce, 2014), thus, leading to increased rumen fermentation and, consequently, methane production. To date, mitigation strategies have focused on identifying low emitting animals, however selection for low methane emitting animals would likely increase the number of days to slaughter thus adding to the producer’s cost while potentially increasing the total methane emissions over the animal’s lifetime. Conversely, selection solely based on reducing the age at finish could lower producer costs but might lead to a larger environmental impact due to higher daily methane emissions. This challenge is further illustrated by Figure 2 which compares age at finish and methane estimated breeding values for animals with GEM system records. Animals were split into quartiles based on the median age at finish EBV and daily methane emissions EBV. Using the average phenotypic length on test and daily methane emissions of the entire group, a total emissions phenotype was derived using the deviations in the breeding values. Animals in the combined selection group (Reduced Age at Finish and Low Methane emissions) will emit an average of 1.02kg less methane (19.19kg vs. 18.17kg) during the finishing period compared to animals selected solely for age at finish (Figure 2). This highlights that the relative emphasis placed on each trait within the index is crucial to ensuring that both profitability and environmental targets can be achieved simultaneously. As speculation around the introduction of carbon taxes grows (Wirsenius et al., 2011; Slade, 2018; Alvim and Sanguinet, 2021; Coderoni et al., 2024), these correlations highlight the trade-offs producers face when balancing environmental goals with economic viability. This furthermore raises questions about how emissions taxes would be applied—whether they would penalize daily emissions or reward overall efficiency. The imposition of a carbon tax could thus shape breeding and management decisions, influencing whether farmers prioritize short-term reductions or long-term sustainability in methane output.

### Carbon dioxide

Whilst methane often dominates the conversation, carbon dioxide also has significant global warming potential (European Commission, 2020). Heritability estimates for carbon dioxide in the present study ranged from moderate to strong (0.24 to 0.50) (Table 1), similar to those previously reported by Donoghue et al. (2020), van Breukelen et al. (2022) and Ryan et al. (2024). Genetic correlations between carbon dioxide and all terminal index traits are reported in Table 5. Unlike methane, all correlations between carbon dioxide and calving difficulty traits were positive, suggesting that carbon dioxide production was associated with increased incidence of calving difficulty (Table 5). Gestation length was moderately and negatively correlated with carbon dioxide (−0.23 to −0.24) whilst mortality had a moderate, positive correlation with carbon dioxide (0.36 to 0.38). These results suggest that selection for reduced carbon dioxide emissions would result in animals with longer gestation and decreased mortality. Reduced gestation lengths in Irish seasonal production systems is economically beneficial to producers as shorter gestation results in a longer effective breeding season and reduces barren rates which leads to involuntary culling, (Amer et al., 1996), and by increasing weaning weights by increasing the suckling period (Crosson et al., 2024). Similar to that exhibited with methane, docility had a moderate positive correlation (0.17 to 0.25) with carbon dioxide indicating that more docile animals produce more carbon dioxide.

**Table 5.** Genetic correlations between carbon dioxide traits and economically important traits in a finishing system, with standard error (SE) in parenthesis

Index Trait		Carbon Dioxide Trait				
		Spot measure (SE)	1-d average (SE)	5-d average (SE)	10-d average (SE)	Full test average (SE)
Calving traits	Gestation	−0.23^**^ (0.065)	−0.24^**^ (0.083)	−0.23^**^ (0.082)	−0.24^**^ (0.081)	−0.23^**^ (0.083)
	Mortality	0.38^*^ (0.117)	0.37^**^ (0.139)	0.36^**^ (0.138)	0.37^**^ (0.137)	0.38^**^ (0.138)
	Primiparous calving difficulty	0.18 (0.146)	0.06 (0.172)	0.05 (0.171)	0.04 (0.170)	0.04 (0.175)
	Multiparous calving difficulty	0.08 (0.115)	0.04 (0.135)	0.03 (0.134)	0.03 (0.134)	0.03 (0.136)
	Docility	0.17^*^ (0.051)	0.25^*^ (0.064)	0.25^*^ (0.063)	0.25^*^ (0.064)	0.23^*^ (0.064)
Efficiency traits	Feed intake	0.88^*^ (0.030)	0.76^*^ (0.063)	0.75^*^ (0.063)	0.75^*^ (0.063)	0.87^*^ (0.028)
	Age at finish	−0.46^*^ (0.055)	−0.55^*^ (0.070)	−0.56^*^ (0.068)	−0.44^*^ (0.075)	−0.50^*^ (0.074)
Carcass traits	Carcass weight	0.71^*^ (0.034)	0.82^*^ (0.033)	0.81^*^ (0.034)	0.81^*^ (0.035)	0.82^*^ (0.039)
	Carcass conformation	0.25^*^ (0.043)	0.35^*^ (0.056)	0.34^*^ (0.055)	0.36^*^ (0.058)	0.35^*^ (0.060)
	Carcass fat	0.39^*^ (0.043)	0.47^*^ (0.052)	0.47^*^ (0.051)	0.47^*^ (0.051)	0.45^*^ (0.053)

Significance levels are denoted as follows: ^*^P < 0.001, ^**^P < 0.01, ^***^P < 0.05.

Carbon dioxide was positively correlated with all carcass traits and feed intake, corroborating previous results by Donoghue et al. (2020) and suggesting that animals with higher feed intakes and animals with heavier carcass weights produce more carbon dioxide (Table 5). The strong correlation between carbon dioxide and feed intake (0.75 to 0.88) in the present study also suggests the opportunity for feed intake to be used as a proxy for carbon dioxide or vice versa (Donoghue et al., 2020). However, further research is needed to assess the viability of this approach, as factors beyond feed intake, such as rumen efficiency or metabolic rate, may attribute to carbon dioxide production (Zaman et al., 2021). This is further supported by the residual correlation with feed intake ranging from 0.16 to 0.20 across carbon dioxide trait definitions, which suggests that a proportion of the relationship between carbon dioxide and feed intake remains unexplained by the current model (Figure S1).

### Balancing breeding objectives

Rowe et al. (2019) highlighted the importance of carefully assessing genetic correlations between methane emissions and production traits to ensure that breeding programs effectively achieve their overarching objectives without compromising genetic gain. The importance of understating the relationship between methane and production is further substantiated by the potential for negative unintended consequences (Pritchard et al., 2013), such as selection for high milk yield had on reducing fertility and increasing metabolic disorders in dairy cows (Oltenacu and Broom, 2010), and rapid growth had on skeletal and cardiovascular issues in broilers (Julian, 1998). Although it is not clear what impact selection for low methane emitters has on animal health, Bain et al. (2014) found clear rumen differences between sheep identified as high or low methane emitters, which could potentially negatively impact feed intake and digestive efficiency. This demonstrates the complex trade-offs involved in targeted genetic selection for reduced emissions whilst maintaining a profitable beef system.

As evidenced in the present study, the current terminal breeding goal is associated with an increase in both methane and carbon dioxide across several economically important traits in beef finishing systems. However, this study focused on gross methane emissions per day, whereas many previous studies have investigated alternative methane traits which often had a production element captured in their definition. Manzanilla-Pech et al. (2022) analyzed a gross methane production trait, alongside alternative methane definitions such as methane intensity (methane divided by energy corrected milk), methane yield (methane divided by dry matter intake) and residual methane (residual from the partial regression of methane on energy corrected milk and metabolic liveweight). Manzanilla-Pech et al. (2022) demonstrated the benefits of using a residual methane trait when selecting for reduced methane emissions in dairy cattle, as residual methane is strongly correlated with efficiency traits such as residual feed intake, and weakly correlated with production traits such as energy corrected milk. Additionally, residual methane and gross methane had a strong genetic correlation (0.82, SE= 0.07; Manzanilla-Pech et al. (2022)), suggesting they could be treated as being the same trait. This suggests that selection on residual methane would reduce gross methane, without negatively impacting production. In contrast, the present study indicates that selection for reduced methane emissions indirectly through correlated traits such as feed intake would not be possible in Irish production systems, as the genetic correlation is less than 0.8.

Despite the existence of antagonistic genetic correlations among some traits (e.g. methane and carcass performance), genetic improvement in all traits remains achievable once the antagonistic genetic correlations are less than unity (Berry, 2015). However, because of these genetic antagonisms, the ideal performance of each trait may not be achievable and consideration must be given to the relative emphasis placed on the trait then within the breed goal to achieve the desired genetic gains. Furthermore, to facilitate the development of accurate methane genetic evaluations and, thus, genetic gain, a large number of accurate methane phenotypic records must be collected (de Haas et al., 2017) and ideally these records should be collected from an environment reflective of the breeding goal. The phenotypes in the present study were collected from controlled indoor environments and high input systems on growing animals, which is not reflective of the seasonal grass-based production system that is predominately practiced in Ireland for a large proportion of a meat destined animal’s life (Hickey et al., 2022; Ryan et al., 2024) and therefore does not reflect methane output across the full growth trajectory of an animal throughout its’ lifetime. As methane recording initiatives expand globally, accumulation of methane data particularly in systems reflective of more common production systems such as grass-based systems, will be paramount to the success of breeding programmes focused on methane reduction.

### Methane trait definition

Internationally there has been a lack of consensus on the best methane trait definition for incorporation of methane into breeding goals (de Haas et al., 2017; Lassen and Difford, 2020). Furthermore, there has been a paucity of studies examining the relationship between multiple methane trait definitions and traits of economic importance in finishing systems. The consistent correlations across gross daily methane trait definitions in the present study suggests that the specific methane trait definition is less critical than the overall breeding strategy. However, the observed variation in the strength of these relationships, particularly with feed intake, highlights the need for a nuanced approach. Notably, evaluating each individual spot measure in a repeatability based model consistently exhibited the lowest standard error in its correlations with the other traits, indicating greater reliability in these estimates. This suggests that a repeated records model using spot measure emission traits may be particularly valuable for accurately assessing genetic relationships in future breeding programs. Nevertheless, as previously highlighted by Ryan et al. (2024) a considerable number of animals are required to achieve accurate predictions; a calibration population of 7,390 animals would be required to generate genomic predictions with 0.70 accuracy whereas 32,767 animals would be needed to achieve 0.90 accuracy using the spot measure methane definition. Given the relatively small standard errors observed in this study, the results suggest that spot measure traits may provide reliable estimates with fewer records, but a cost-benefit analysis is essential to determine whether the additional expense of phenotyping larger populations is justified by the gains in prediction accuracy, before establishing the most appropriate methane trait for inclusion in the breeding goal.

## Conclusion

The results of this study highlight the intricate relationship between gross daily methane emissions and production traits related to beef finishing systems. Selecting for improved efficiency is challenging, as focusing solely on reducing daily methane emissions could unintentionally lead to smaller carcass weights, delayed finishing, and negative impacts on overall animal productivity. Nevertheless, the parameters and relationships quantified in this study offer a foundation for generating estimated breeding values for methane and constructing a balanced breeding index tailored to finishing systems. This approach would help mitigate unintended negative consequences, ensuring that reducing methane emissions does not compromise important economic traits like feed intake and carcass quality.

## Supplementary Data

Supplementary data are available at *Journal of Animal Science* online.

skaf162_suppl_Supplementary_Tables_S1-S3

## Abbreviations:

AI: artificial insemination
CV: coefficient of variation
EBV: estimated breeding value
GEM: GreenFeed Emission Monitoring
ICBF: Irish Cattle Breeding Federation
