# Supplementary material for: Genetic correlations between enteric methane and traits of economic importance in a beef finishing system
Source: J Anim Sci. 2025 May 13;103:skaf162. doi: 10.1093/jas/skaf162 (PMC12132798; doi:10.1093/jas/skaf162)
Supplement: skaf162_suppl_Supplementary_Tables_S1-S3 [file skaf162_suppl_supplementary_tables_s1-s3.docx]

**Supplementary materials**

Table S1. Breed breakdown of 1,794 animals with GEM system measurements

| Animal Type | Beef x Beef | | Dairy x Beef | | Dairy | |
| --- | --- | --- | --- | --- | --- | --- |
| Number of intakes | 35 | | 18 | | 9 | |
| Number of animals | 992 | | 434 | | 368 | |
| Average Breed % | Sire breed | Dam breed | Sire breed | Dam breed | Sire breed | Dam breed |
| Angus | 12.3% | 13.2% | 41.9% | - | - | - |
| Aubrac | 5.2% | 0.8% | 8.5% | - | - | - |
| Belgian blue | 2.6% | 3.8% | 3.0% | - | - | - |
| Charolais | 22.8% | 12.2% | 0.7% | - | - | - |
| Holstein/Friesian | - | 0.6% | - | 89.9% | 80.1% | 85.1% |
| Hereford | 2.5% | 5.9% | 28.3% | - | - | - |
| Jersey | - | - | - | 4.8% | 18.5% | 13.0% |
| Limousin | 28.8% | 33.8% | 15.0% | - | - | - |
| Piedmontese | 1.4% | 1.9% | - | - | - | - |
| Parthenaise | 2.2% | 1.3% | - | - | - | - |
| Saler | 6.6% | 4.3% | - | - | - | - |
| Shorthorn | 3.5% | 1.8% | 1.1% | - | - | - |
| Simmental | 10.8% | 18.8% | - | - | - | - |
| Other | 1.2% | 1.6% | 1.4% | 5.3% | 0.5% | 1.9% |

Table S2. Breakdown of the Terminal Index

|  | Trait | Relative Emphasis | Description | Direction of Selection |
| --- | --- | --- | --- | --- |
| Calving | Primiparous calving difficulty* | 12 | Percentage of births in beef heifers requiring considerable assistance and /or veterinary assistance. | **↓** |
|  | Multiparous calving difficulty* |  | Percentage of births in beef cows requiring considerable assistance and /or veterinary assistance. | **↓** |
|  | Gestation* | 4 | The number of days between a known conception date and a subsequent calving date. | **↓** |
|  | Mortality* | 4 | Mortality rate is a measure of the number of deaths (in general, or due to a specific cause, i.e. associated with calving, within 5 days of birth). | **↓** |
| Carcass | Carcass Weight* | 33 | Carcass Weight is defined as the weight of both half carcasses after being bled, eviscerated and after removal of skin, removal of external genitalia, the limbs at the carpus and tarsus, head, tail, kidneys and kidney fats and the udder. | **↑** |
|  | Carcass Conformation* | 7 | Carcass Conformation is the shape and development of the carcass. It is denoted by the letters E, U, R, O, P with E being the best and P the poorest and subsequently divided into a 15-point scale with the use of +, =, and – for each letter grade. | **↑** |
|  | Carcass Fat* |  | Carcass Fat is the level of fat covering on the carcass. It is denoted by a scale from 1 to 5 (1 being lean and 5 being fattest) and subsequently divided into a 15-point scale with the use of +, =, and – for each fat score. | **↑** |
|  | Factory Specs | 7 | Percentage likelihood of meeting abattoir carcass specifications based on carcass weight breeding value, carcass conformation breeding value and carcass fat breeding value. |  |
| Efficiency | Age at Finish* | 7 | Age at finish is the age at which an animal reaches appropriate condition for slaughter. | **↓** |
|  | Feed Intake* | 11 | Feed Intake is the amount of feed consumed by a young growing animal for the duration of the finishing period. | **↓** |
| Other | Docility* | 1 | Docility describes the way in which an animal behaves, with regard to humans, other animals and during specific activities such as calving or feeding. | **↑** |
|  | Polled | 2 | Euro value bonus applied to polled animals, reflective of the labour saving due to progeny being polled. |  |
|  | AA/HE Bonus | 6 | Euro value bonus applied to purebred Angus and Hereford male animals, reflective of the bonus their progeny receive from the abattoirs at slaughter. |  |
|  | Carbon | 6 | A sub index comprising of economic weights applied to traits which impact carbon emissions of the animal, under an assumed carbon pricing of €80/ton. |  |

* denotes traits included in the bivariate analysis in this study.

Table S3. Genetic and residual correlations (± standard error) between feed conversion efficiency and emission traits, calculated across five methane and carbon dioxide trait definitions.

| **Trait Definition** | **Methane** | | **Carbon Dioxide** | |
| --- | --- | --- | --- | --- |
|  | **Genetic Correlation** | **Residual Correlation** | **Genetic Correlation** | **Residual Correlation** |
|  | **(SE)** | **(SE)** | **(SE)** | **(SE)** |
| Spot measure | -0.21 | -0.09 | 0.05 | -0.14 |
|  | (0.156) | (0.004) | (0.138) | (0.004) |
| One day average | -0.19 | -0.15 | 0.04 | -0.17 |
|  | (0.157) | (0.004) | (0.147) | (0.004) |
| 5-day average | -0.16 | -0.16 | 0.08 | -0.26 |
|  | (0.159) | (0.008) | (0.147) | (0.008) |
| 10 day average | -0.17 | -0.19 | 0.07 | -0.28 |
|  | (0.158) | (0.012) | (0.147) | (0.011) |
| Full test average | -0.21 | -0.15 | 0.02 | -0.12 |
|  | (0.159) | (0.012) | (0.149) | (0.014) |


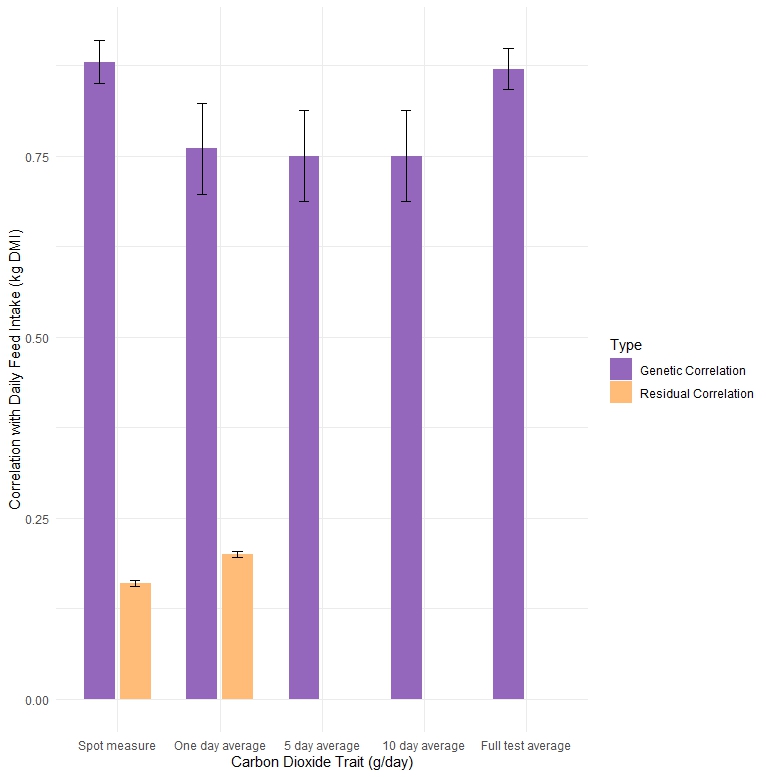


Figure S1. Genetic and residual correlations between daily feed intake and carbon dioxide traits with error bars. Error bars represent the standard error of correlation coefficients.
